# Correlation between Water Characteristics and Gel Strength in the Gel Formation of Golden Pompano Surimi Induced by Dense Phase Carbon Dioxide

**DOI:** 10.3390/foods12051090

**Published:** 2023-03-03

**Authors:** Weiwen Duan, Hui Qiu, Kyi Kyi Htwe, Zefu Wang, Yang Liu, Shuai Wei, Qiuyu Xia, Qinxiu Sun, Zongyuan Han, Shucheng Liu

**Affiliations:** 1 Guangdong Provincial Key Laboratory of Aquatic Product Processing and Safety, Guangdong Province Engineering Laboratory for Marine Biological Products, Guangdong Provincial Engineering Technology Research Center of Seafood, Key Laboratory of Advanced Processing of Aquatic Product of Guangdong Higher Education Institution, College of Food Science and Technology, Guangdong Ocean University, Zhanjiang 524088, China; 2 Guangdong Laboratory of Southern Marine Science and Engineering (Zhanjiang), Zhanjiang 524088, China; 3 Collaborative Innovation Center for Key Technology of Marine Food Deep Processing, Dalian University of Technology, Dalian 116034, China

**Keywords:** golden pompano, surimi processing, dense phase carbon dioxide, gel strength, water characteristics

## Abstract

The relationship between the gel quality of golden pompano surimi treated with dense phase carbon dioxide (DPCD) and changes in water characteristics was evaluated. Low-field nuclear magnetic resonance (LF-NMR) and nuclear magnetic resonance imaging were used to monitor changes in the water status of surimi gel under different treatment conditions. Whiteness, water-holding capacity and gel strength were used as the quality indicators of the surimi gel. The results showed that DPCD treatment could significantly increase the whiteness of surimi and the strength of the gel, while the water-holding capacity decreased significantly. LF-NMR analysis showed that, as the DPCD treatment intensity increased, the relaxation component *T*_22_ shifted to the right, *T*_23_ shifted to the left, the proportion of *A*_22_ decreased significantly *(p* < 0.05*)* and the proportion of *A*_23_ increased significantly *(p* < 0.05*)*. A correlation analysis of water characteristics and gel strength showed that the water-holding capacity of surimi induced by DPCD was strongly positively correlated with gel strength, while *A*_22_ and *T*_23_ were strongly negatively correlated with gel strength. This study provides helpful insights into the quality control of DPCD in surimi processing and also provides an approach for the quality evaluation and detection of surimi products.

## 1. Introduction

Golden pompano (*Trachinotus ovatus*) is one of the marine fishes with important economic value in the southern coastal region of China. It is popular with consumers for its tender flesh, delicious taste and high content of polyunsaturated fatty acids [1]. China is one of the world’s largest producers of golden pompano, with a total domestic mariculture production of 243,900 tonnes in 2021; this ranked second among all marine economic fish and was an increase of 139.85% compared to 2020 [2].

Currently, golden pompano is mainly sold as fresh and frozen fish. Surimi is an important processed aquatic product, which is mainly formed by heat induction. However, surimi processed by heat induction is prone to the loss of heat-sensitive nutrients due to excessive temperatures. Additionally, in the process of heat-induced surimi formation, the surimi remains at 50–70 °C for long periods due to the slow heating rate and heat transfer rate of heat induction. This is the optimum temperature for the enzymatic activity of the alkaline protease in the surimi gel, resulting in the degradation of myofibrillar protein and leading to gel deterioration, which in turn results in the deterioration of surimi quality [3,4]. High-density CO_2_ (dense phase carbon dioxide, DPCD) is a new non-thermal processing technology that generally operates at temperatures (<60 °C) and pressures (<50 MPa). A high-pressure acidic environment is created by the pressure of the carbon dioxide and its molecular action. Unlike conventional heat treatment, DPCD technology can be used at mild processing conditions, which can be applied in sterilisation, enzyme inactivation and promotion of food quality attributes such as textural and nutritional properties [5]. In addition, CO_2_ has a low viscosity and high diffusivity, which enables it to penetrate the bacterial cell membranes. The advantage of this method can avoid thermal damage and maintain food quality [6,7]. It has been shown that DPCD treatment can denature proteins to form gels with significantly better gel characteristics than those of traditional heat-induced gels while preserving the nutritional value and flavour of the food to the greatest extent possible; thus, DPCD can be used as an alternative to traditional thermal processing methods [8].

Gel strength and water-holding capacity are important quality characteristics of surimi. During processing, surimi proteins stretch and denature to form a three-dimensional network structure that holds more water [9]. Generally, surimi gel has a high water content. The distribution of water in surimi gel and the relative proportions of water in different states will directly affect the quality of surimi gel [10]. In this study, the effects of different DPCD treatment conditions on the water characteristics of golden pompano surimi gel were compared. The water distribution and relative proportions in surimi gel under different treatment conditions were investigated by low-field nuclear magnetic resonance (NMR) spectroscopy. The relationships between the gel strength, water-holding capacity and whiteness of the surimi gel were investigated in relation to its water characteristics. This work aimed to reveal the quality changes relating to DPCD-induced gel formation in golden pompano surimi from the perspective of water characteristics and gel strength. This work also provides a theoretical basis for evaluating and improving the quality of DPCD-induced gel formation in golden pompano surimi.

## 2. Materials and Methods

### 2.1. Chemicals

Pure Carbon dioxide (99.99%) was obtained from the Zhanjiang Oxygen Plant. Sucrose and sodium chloride were purchased from Guangdong Guanghua Sci-Tech Co., Ltd. (Guangzhou, China). All reagents were analytical grade.

### 2.2. Surimi Sample Preparation

Golden pompano fish of average weight (750 ± 50 g) were purchased from Dongfeng Seafood Market (Zhanjiang, China). The fish was kept in oxygenated water and immediately transported to the laboratory within 1 h. Then, the fish were rapidly immersed in ice water and the surimi was prepared according to the method proposed by Liu et al. [11].

The fish were rinsed, scaled, headed, gutted and cleaned to remove any remaining offal from the abdominal cavity, keeping the water temperature below 10 °C during the cleaning process. The fish flesh was then placed in a roller-type flesh separator (SZC-180, Jinan, China) to separate the flesh. The resulting minced fish was rinsed in five batches of ice water three times (10 min/each).

**Figure 1:**
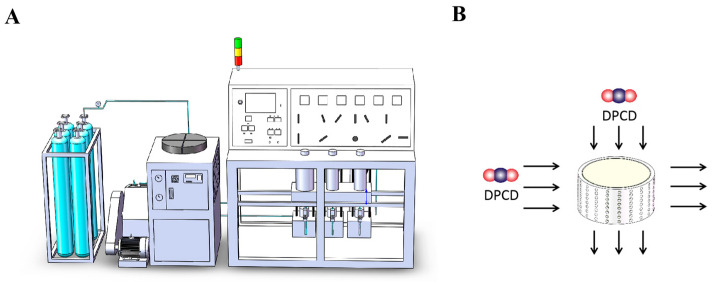
DPCD processing unit diagram (**A**) and mold drawing (**B**): colorful balls is dense phase carbon dioxide.

Next, the minced fish was squeezed and dewatered in three layers of gauze and then dehydrated in a minced fish spiral dehydrator (ZTZY-120, Xinxiang, China) at 30 rpm for 3 min. The minced fish was supplemented with 1% sucrose by mass and chopped with a blender (MQ785, Germany Braun Co. Ltd., Cluj-Napoca, Romania) at 14,200 rpm for 5 min. Next, the minced fish was placed in a custom-made cylindrical mold (Figure 1B) and used for DPCD or heat treatment.

### 2.3. Experimental Design

Based on our previously reported research method [5], a schematic diagram of the DPCD treatment equipment is shown in Figure 1A. The temperature of the DPCD treatment kettle was set prior to the experiment and the sample was not inserted until the kettle had been heated to the set temperature. The surimi was formed into a cylinder (thickness 20 mm, diameter 40 mm) using a mold (Figure 1B), placed in the DPCD treatment kettle and sealed. The CO_2_ inlet and outlet valves were opened for 30 s to evacuate the air from the kettle and then the outlet valve was closed. Next, the pressure was kept constant when the CO_2_ pressure reached the target condition in the sample-treated kettle. After the treatment was completed, the exhaust valve was opened to slowly depressurise the kettle. Then, the surimi gel was removed and packaged in a sealed bag. The test indices were measured after 12 h at 4 °C.

The DPCD treatment group was carried out using a single-factor design. (1) different pressure conditions (5, 10, 15, 20, 25, 30 and 35 MPa) and constant temperature and time (50 °C and 60 min); (2) different temperature conditions (30, 35, 40, 45, 50, 55 and 60 °C) and constant pressure and time (20 MPa and 60 min); and (3) different treatment time (10, 20, 30, 40, 50, 60 and 70 min) and constant pressure and temperature (20 MPa and 50 °C) were applied. The surimi gel quality was tested in each individual factor treatment.

Two control groups were also set up, one for the raw surimi group (Control), which was not treated, and the other for the heat treatment group (WB), which applied two-stage water bath heating (40 °C for 30 min, 90 °C for 30 min).

### 2.4. Determination of Whiteness

The surimi colour parameters were determined using a colourimeter (CR-20, Konica Minolta Inc., Tokyo, Japan). The CIE Lab coordinates were reported as lightness (L*), redness (a*) and yellowness (b*). Whiteness (*W*) was calculated according to Equation (1).

(1) $$W=100-\sqrt{{\left(100-L^{*}\right)}^{2}+{\left(a^{*}\right)}^{2}+{\left(b^{*}\right)}^{2}}$$

### 2.5. Determination of Water-Holding Capacity

The water-holding capacity (WHC) was determined according to the method of Yang et al. [12]. A 5.0 ± 0.1 g gel sample (*m*_1_) was wrapped with filter paper and centrifuged at 4 °C and 5000 rpm for 10 min. Then, the sample was weighed (*m*_2_) after removing the filter paper. The WHC was calculated according to Equation (2).

(2) $$\mathrm{WHC}\;(\%)=\frac{m_{2}}{m_{1}}\times 100$$

### 2.6. Low-Field Nuclear Magnetic Resonance and Magnetic Resonance Imaging

The water distribution was determined using an NMI 20-060H-I NMR analyser (Newmark Analytical Instruments Co., Ltd., Suzhou, China) with a stable frequency of 21.12 MHz at 32 °C. The instrument was calibrated as described by Zhou et al. [13], with a slight modification. TE (echo time) = 0.35 ms; TW (wait time) = 3000 ms; NECH (number of echoes) = 4000 were set. The greyscale image was converted into a colour image using Niumag NMR V3.0 image processing software to visualise the water distribution.

### 2.7. Determination of Gel Strength

The gel strength of the surimi was determined using a TMS-Pro analyser (FTC Co., Ltd., Vienna, Virginia, USA). According to the method described by Zheng et al. [5], a probe = P/0.5 s, trigger force = −5 g; pre-test speed = 5 mm·s^–1^; test speed = 1 mm·s^–1^; compression deformation, 75% were set. The gel strength (g × mm). was obtained by multiplying the breaking strength (g) and the breaking distance (mm).

### 2.8. Statistical Analysis

Experimental data are expressed as the mean ± standard deviation. A variance and Tukey’s HSD multiple comparisons (with a 95% confidence interval) were analysed using JMP 16.0 software. The correlation between water characteristics and gel strength during DPCD-induced gel formation in surimi was analysed via Pearson’s correlation analysis using Origin 2022 software (Origin Lab, Hampton, NH, USA). Three batches of experiments were performed and each batch had three parallel samples.

## 3. Results and Discussion

### 3.1. Effect of Different DPCD Treatments on Surimi Whiteness

The whiteness (*W*) of surimi gel is one of the most important indicators for evaluating its quality. The greater the whiteness, the more favoured the surimi is by consumers. The brightness of an object is related to the light reflectance of its surface, and the intensity of light scattered by the surface of food is closely related to the moisture content of food. In general, the higher the moisture content of the food, the higher the intensity of light scattered from its surface and the greater the whiteness [14,15]. The whiteness of surimi gel is also related to protein denaturation, aggregation and gel networks [16,17]. Generally, the denser the protein gel network structure, the better the water retention and the greater the intensity of light scattering, resulting in greater whiteness.

**Figure 2:**
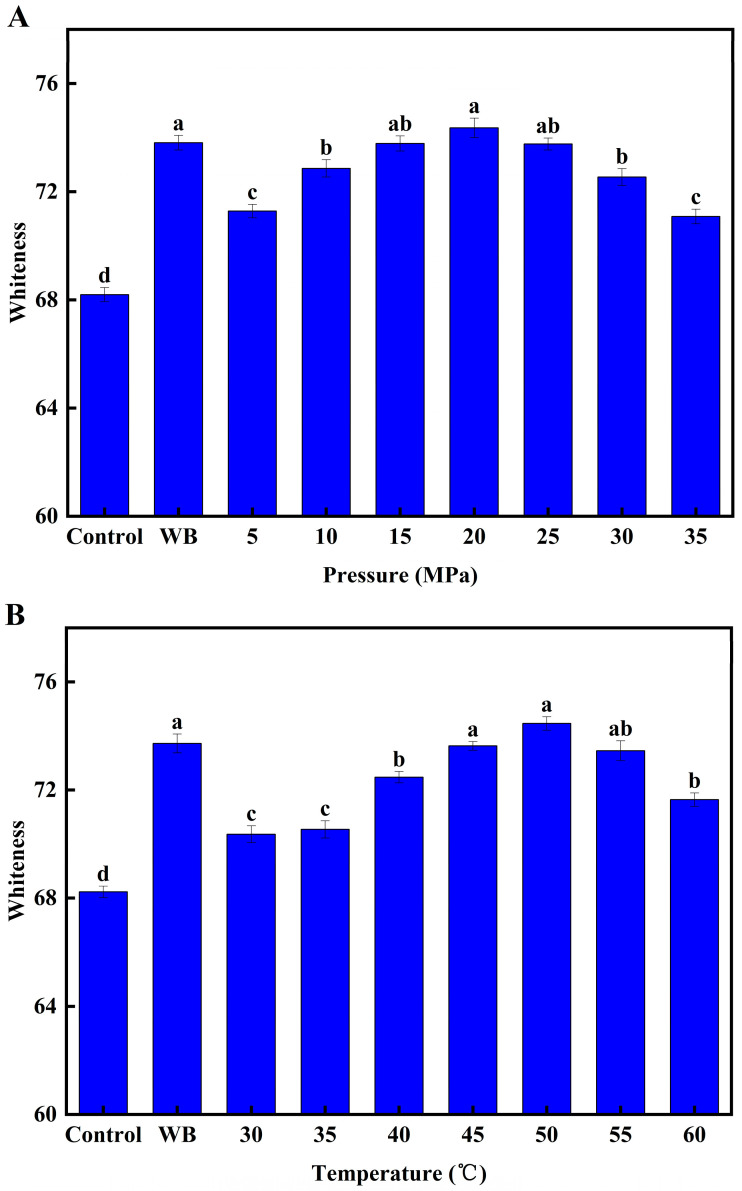

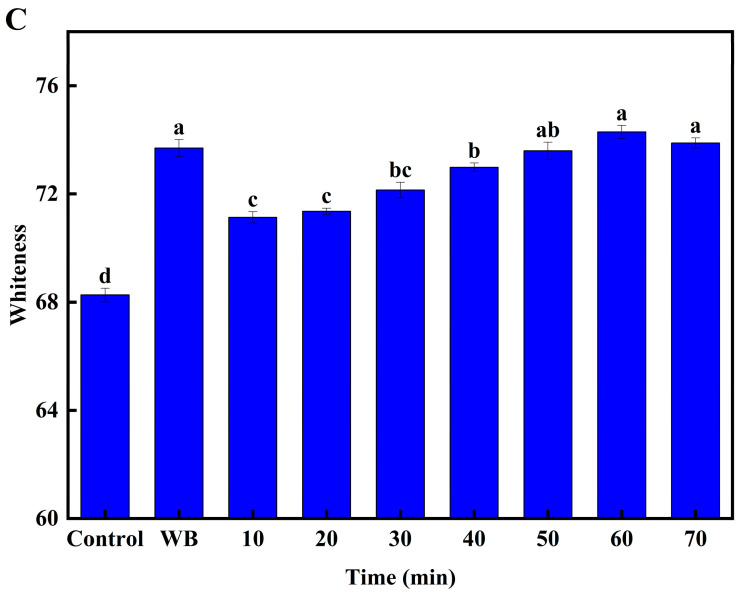
Effect of different DPCD treatments on surimi whiteness (**A**): Pressure, (**B**): Temperature, (**C**): Time. Different letters indicate statistically significant differences (*p* < 0.05).

As shown in Figure 2A–C, both the heat treatment and the DPCD treatment significantly increased the surimi gel whiteness compared to the control group (*p* < 0.05). When the DPCD treatment conditions were 50 °C and 60 min (Figure 2A), the surimi gel whiteness increased with increasing pressure in the range of 5–20 MPa (*p* < 0.05). This was because CO_2_ under pressure caused moderate denaturation and aggregation of proteins, resulting in a dense three-dimensional network structure of the surimi gel to trap moisture. Meanwhile, the whiteness of the surimi gel increased due to increased light scattering. However, the whiteness of the surimi gel decreased when the treatment pressure was higher than 20 MPa (*p* < 0.05). This was because the excessive pressure enhanced the molecular action of CO_2_, resulting in the loss of water from the surimi gel and a decrease in the whiteness of the surimi gel due to the weakening of its light scattering intensity [18].

When the DPCD treatment conditions were 20 MPa and 60 min (Figure 2B), the whiteness of the surimi gel increased as the temperature increased from 30–50 °C (*p* < 0.05). This was because the increase in temperature contributed to the rapid gelation of the surimi. However, when the temperature was 50–60 °C, the surimi gel whiteness decreased as the temperature increased (*p* < 0.05). This was because the high temperature accelerated the evaporation of water, resulting in the loss of water from the surimi gel. The weakening of the light scattering intensity of the surimi gel decreased its whiteness.

The whiteness of the surimi gel increased with time when the DPCD treatment conditions were 20 MPa and 50 °C (Figure 2C) in the range of 10–60 min (*p* < 0.05). This was because increasing the treatment time intensified the surimi gelation. However, when the time was in the range of 60–70 min, there was no significant change in the whiteness of the surimi gel, indicating that surimi gelation was largely complete at 60 min [6].

### 3.2. Effect of Different DPCD Treatments on the Water-Holding Capacity of Surimi

**Figure 3:**
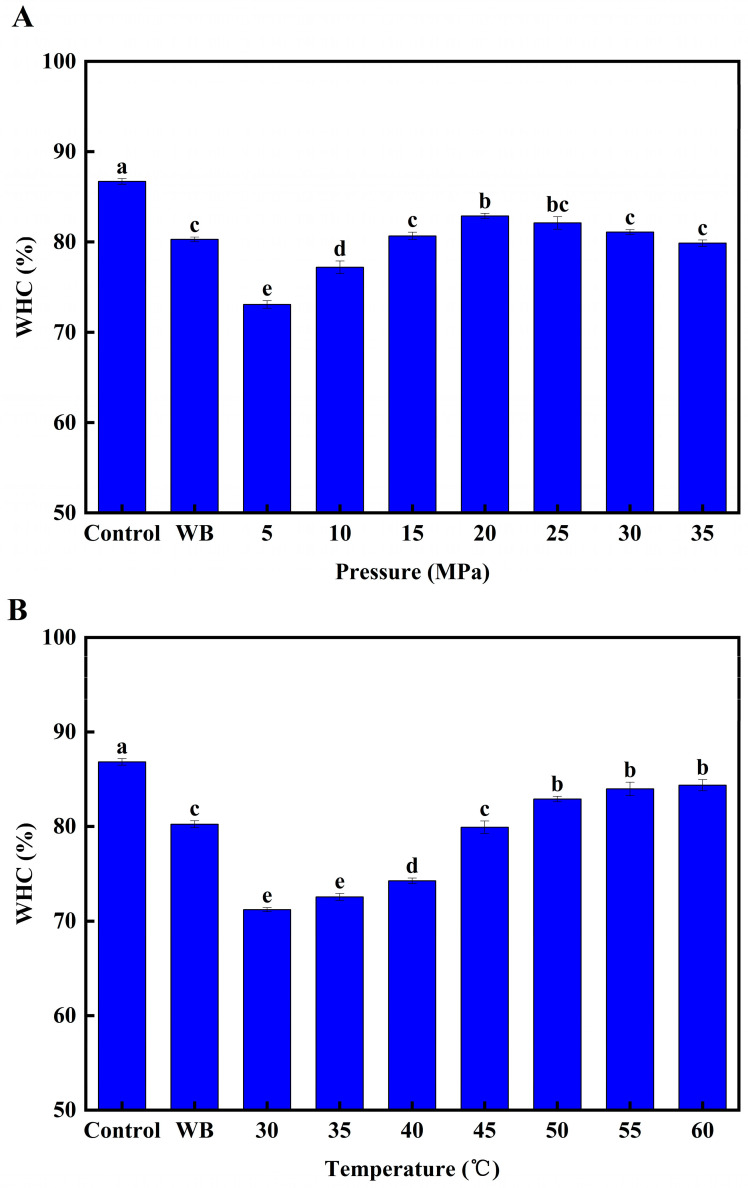

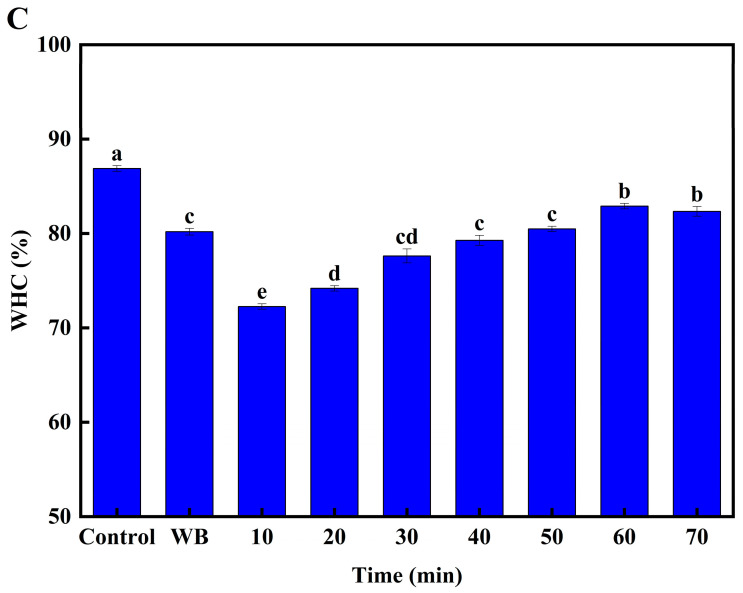
Effect of different DPCD treatments on the water holding capacity of surimi (**A**): Pressure, (**B**): Temperature, (**C**): Time. Different letters indicate statistically significant differences (*p* < 0.05).

Moisture content is a key factor affecting the quality of surimi, accounting for 73–80% of its weight. The amount of moisture will directly affect gel formation [19]. The water-holding capacity (WHC) is an important indicator of the moisture content of surimi and the formation of a gel structure [20]. As shown in Figure 3, the WHC of the surimi gel decreased significantly (*p* < 0.05) in both the heat treatment group and the DPCD treatment group compared to the control group. This was due to the gradual formation of the surimi gel network during the treatment process, which had a binding effect on the water within it [21].

The WHC of the surimi peaked at a treatment pressure of 20 MPa when the DPCD treatment conditions were 50 °C and 60 min (Figure 3A), which was significantly higher than that of the heat-treated samples. When the treatment pressure exceeded 30 MPa, the WHC of the surimi decreased significantly (*p* < 0.05). This could be due to two reasons. First, at 20 MPa the surimi gel formed a dense network structure and thus the WHC was optimal. However, as the treatment pressure continued to increase, the network structure of the surimi gel was destroyed and the WHC decreased. Second, CO_2_ has an extraction effect and during the treatment process, it removed a large amount of water from the surimi.

When the DPCD treatment conditions were 20 MPa and 60 min (Figure 3B) and the temperature was in the range of 30–50 °C, the WHC of the surimi gel increased as the temperature increased (*p* < 0.05). This was because the increase in temperature was conducive to surimi gelation, forming a dense three-dimensional network structure to trap the water. Simultaneously, the increase in temperature enhanced the thermal motion of the CO_2_ molecules, facilitating their penetration and diffusion into the surimi. This also stabilised the gel structure induced by DPCD and was conducive to maintaining the WHC of the surimi gel. In the temperature range of 50–60 °C, there was no significant difference in the WHC of the surimi gel as the temperature increased, indicating that the dense three-dimensional network structure of surimi had essentially formed at 50 °C [6].

When the DPCD treatment conditions were 20 MPa and 50 °C (Figure 3C), and the treatment time was in the range of 10–60 min, the WHC of the surimi gel increased as the treatment time increased (*p* < 0.05). This indicated that there was a cumulative effect of DPCD treatment on the WHC of the surimi gel, with increasing treatment time intensifying the surimi gelation and increasing its WHC. However, when the treatment time reached 60–70 min, there was no significant change in the WHC of the surimi gel, showing that the gelation of surimi was complete by 60 min.

### 3.3. Effect of Different DPCD Treatments on the Water Characteristics of Surimi

#### 3.3.1. Effect of Different DPCD Treatments on the *T*_2_ Relaxation Times of Surimi

**Figure 4:**
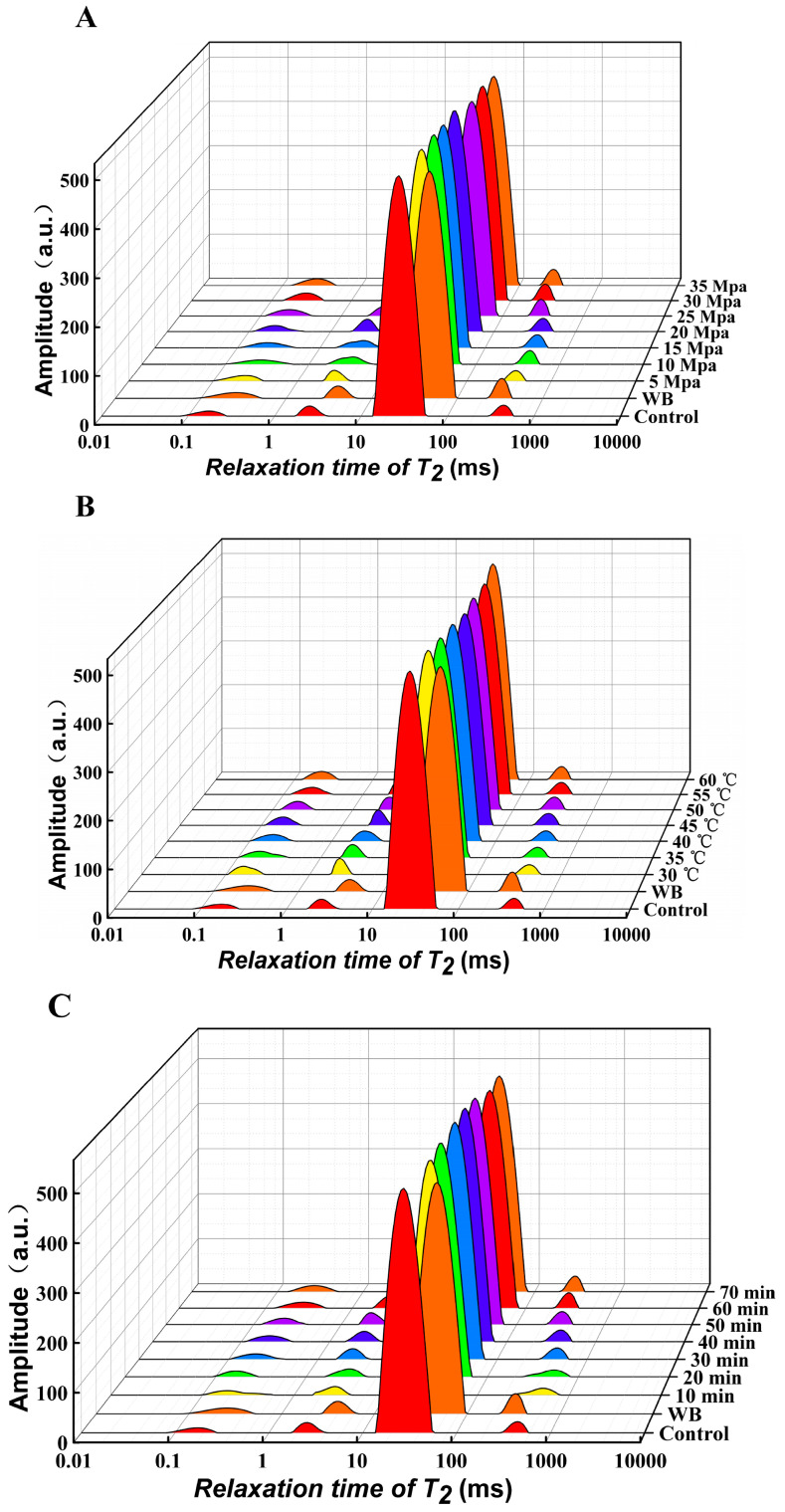
Effect of different DPCD treatments on the *T*_2_ relaxation time of surimi (**A**): Pressure, (**B**): Temperature, (**C**): Time.

The *T_2_* relaxation time reflects the chemical environment of the hydrogen proton, which is related to its degree of freedom and binding force [22]. The shorter the *T*_2_ relaxation time, the greater the binding force or the smaller the degree of freedom of the hydrogen proton in the sample, and the more to the left the peak position on the *T*_2_ inversion spectrum [23]. As shown in Figure 4, there were four peaks in the *T*_2_ inversion spectrum, with the relaxation times represented by *T*_21*a*_, *T*_21*b*_, *T*_22_ and *T*_23_. These correspond to the different states of water molecules in the surimi gel: bound water (*T*_21*a*_, *T*_21*b*_; 0.01–10 ms), immobilised water (*T*_22_; 30–100 ms) and free water (*T*_23_; >1000 ms) [24].

There was a tendency for *T*_21*a*_ and *T*_21*b*_ to shift to the right as the intensity of the DPCD treatment increased, compared to the control group, and their relaxation time range increased. This could be one reason for the significant decrease in the WHC for each group of DPCD treatment samples compared to the control group (Figure 3). Conversely, as the DPCD intensity increased, the protein conformation of the surimi changed and the exposure of hydrophobic groups affected the hydration of the protein, resulting in an increase in the degree of freedom (*T*_21*a*_ and *T*_21*b*_) [8]. However, as the DPCD intensity increased, *T*_22_ tended to shift to the right, indicating an increase in the relaxation times of this part of the water, which reflected the macromolecular water retained in the gel. During the treatment process, the myofibrillar protein was denatured with the penetration and diffusion of CO_2_, which changed the state of the macromolecular water that was retained in the three-dimensional network of the gel. Thus, the degree of freedom of *T*_22_ increased significantly during the treatment process, which may be a major reason for the significant decrease in the WHC of the samples in each treatment group shown in Figure 3 [25]. In contrast, *T*_23_ tended to move to the left as the DPCD treatment intensity increased. This indicated that the degree of freedom of free water in the surimi gradually decreased under the effect of DPCD treatment. This was due to the gradual contraction of the myofibrillar protein after denaturation to form a network structure, resulting in changes in the environment of this part of the water with the highest degree of freedom. At the same time, the gel network structure formed a certain binding capacity for free water during the treatment [7].

#### 3.3.2. Effect of Different DPCD Treatments on the Different States of Water Content in Surimi

The signal intensity of the *T*_2_ inversion spectrum is positively correlated with the moisture content of the sample components. Accordingly, the areas of the four peaks were calculated for each group of samples in Figure 4. The area of each peak was then expressed as a percentage of the total area, representing the percentage of moisture content in each of the different states (*A*_21*a*_, *A*_21_*_b_*, *A*_22_ and *A*_23_) [26].

**Figure 5:**
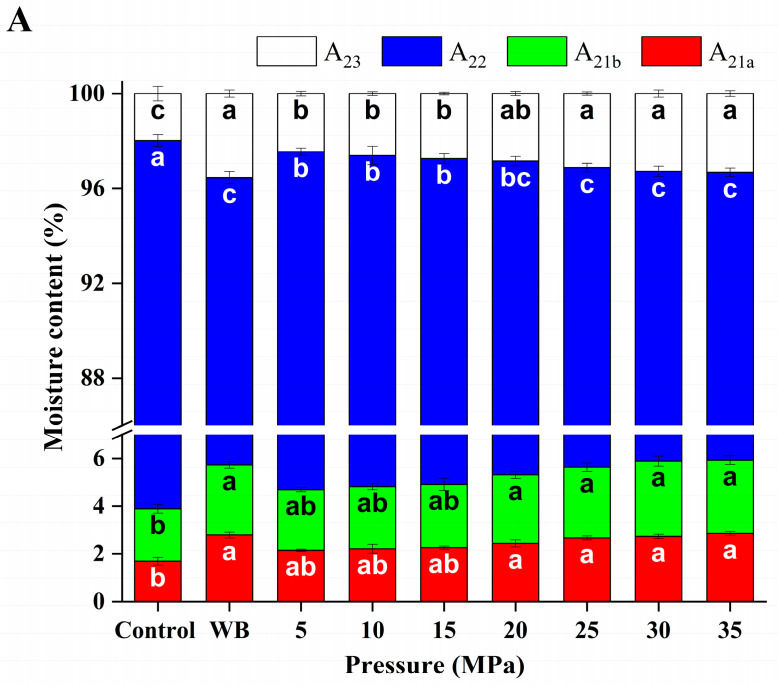

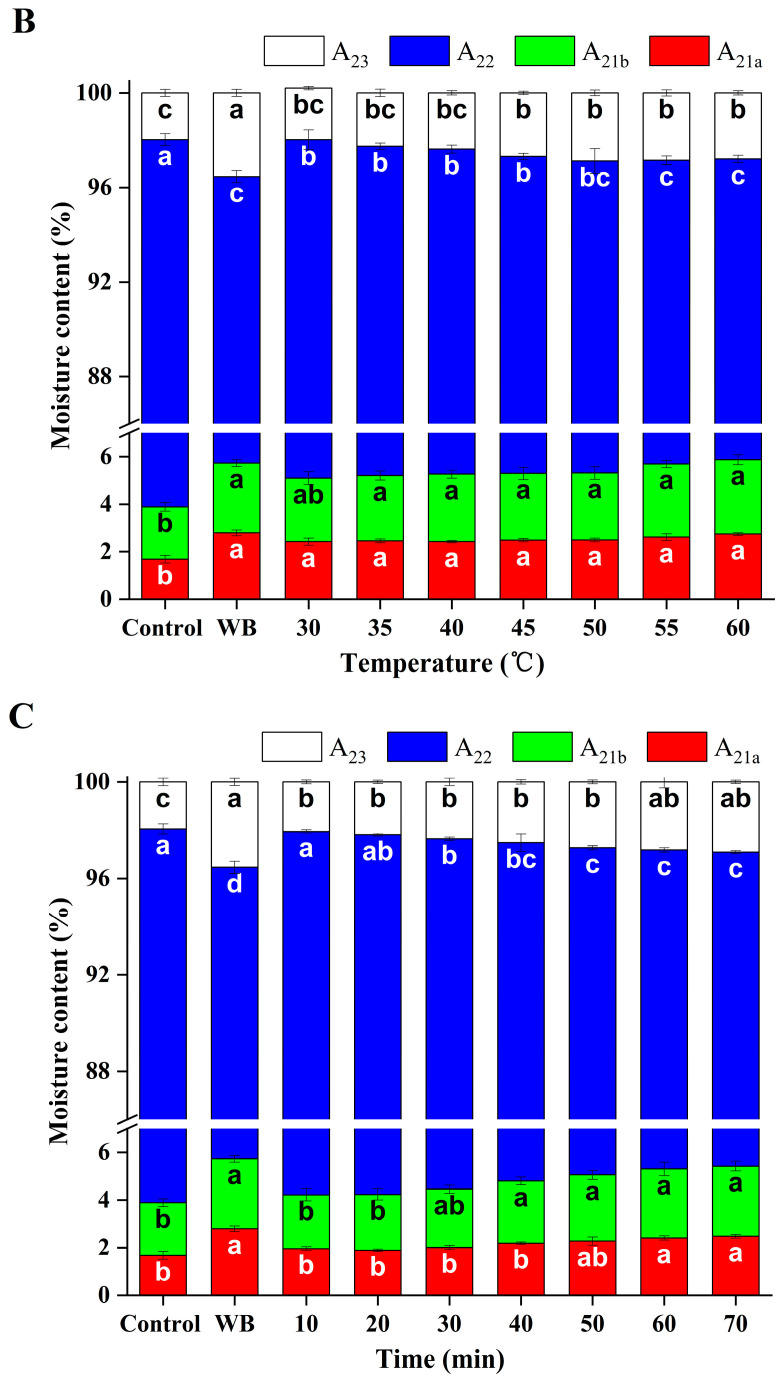
Effect of different DPCD treatments on the different states of the water content in surimi (**A**): Pressure, (**B**): Temperature, (**C**): Time. Different letters indicate statistically significant differences (*p* < 0.05).

As shown in Figure 5, the highest moisture content in the surimi gel was *A*_22_. Compared with the control group, *A*_22_ decreased significantly (*p* < 0.05) and *A*_23_ increased significantly (*p* < 0.05) after DPCD treatment. During the DPCD treatment, the myofibrillar proteins in the surimi were denatured by the combined effects of CO_2_, pressure and temperature. The advanced structure was loosened, the peptide chains stretched and the water binding force was weakened. Then, the molecules aggregated and cross-linked to form a gel network with hydrogen bonds, hydrophobic interactions, non-disulphide covalent bonds, disulphide bonds, etc. This resulted in the conversion of immobile water into free water, leading to a significant decrease in *A*_22_ and a significant increase in *A*_23_ (*p* < 0.05) [27,28]. There was no significant change in *A*_23_ as the intensity of the DPCD treatment increased (>30 MPa, >50 °C, >60 min). This was because *A*_23_ was extruded by DPCD while immobile water became free water, and the formation of a gel network bound a certain content of water so that the migration of the two water fractions reached equilibrium [8]. Additionally, the proportion of *A*_21*a*_ and *A*_21*b*_ also increased significantly (*p* < 0.05), which may have been because DPCD treatment mainly caused the loss of free water and immobile water, while the content of bound water did not change; thus, the proportion of bound water increased.

#### 3.3.3. Effect of Different DPCD Treatments on Magnetic Resonance Imaging of Surimi

**Figure 6:**
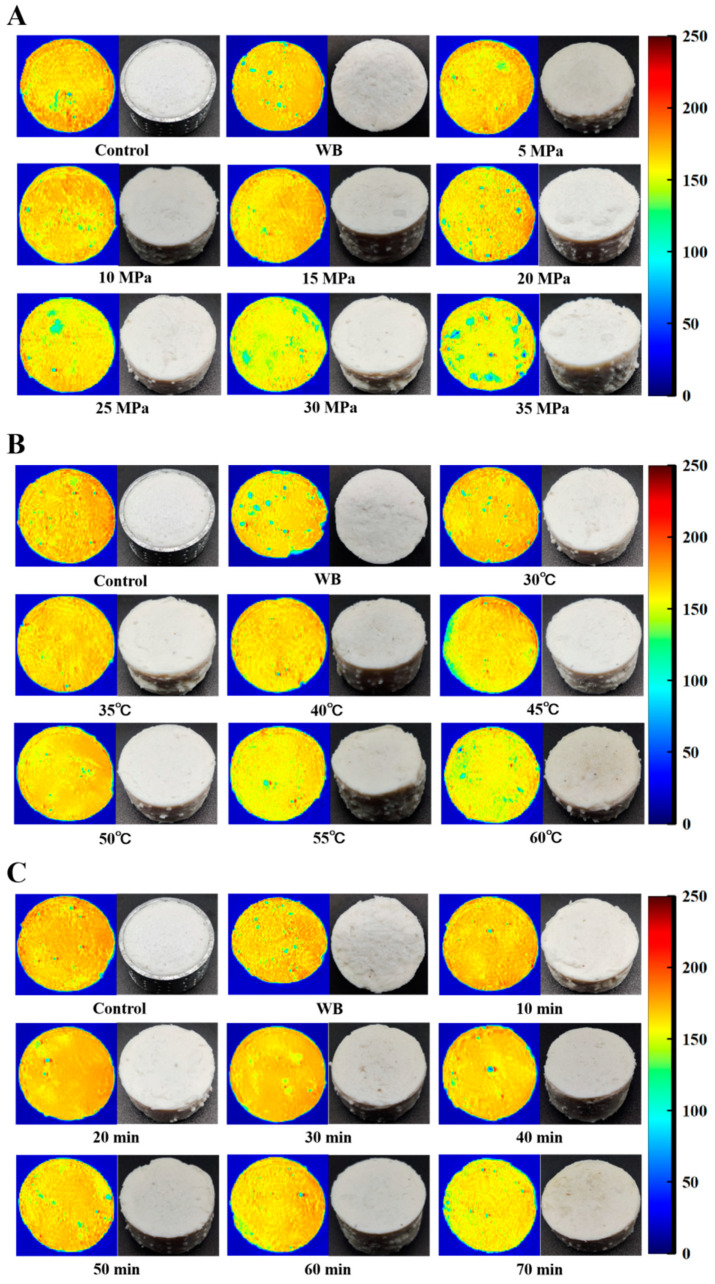
Effect of different DPCD treatments on MRI of surimi (**A**): Pressure, (**B**): Temperature, (**C**): Time.

Magnetic resonance imaging (MRI) can be used to visualise the moisture distribution and migration of food. Figure 6 shows the changes in the MRI images of surimi under the different DPCD treatments; red indicates a higher hydrogen proton density and higher moisture content, while blue indicates a lower hydrogen proton density and lower water content [29,30]. The MRI images of the surimi show that the red parts gradually decreased as the DPCD processing intensity increased, corresponding to a weakening of the relaxation signal. This indicated that the hydrogen proton density in surimi gradually decreased and water was continuously lost as the treatment intensity increased. This further confirmed the results of the change in moisture content shown in Figure 5.

### 3.4. Effect of Different DPCD Treatments on the Surimi Gel Strength

Gel strength refers to the force per unit area of a gel when it disintegrates or breaks; it corresponds to the tightness of the internal structure of the gel, which is related to the denaturation of the protein [31]. Our previous study found that surimi could be induced to form a gel by DPCD treatment [5,9] through two main effects. First, the CO_2_ in DPCD dissolved in water to form carbonic acid, which in turn dissociated to form H^+^, HCO_3_^−^ and CO_3_^2–^ ions, decreasing the pH of the system. Then, interactions with proteins occurred through hydrogen bonding and protonation. Protein molecules interacted with each other through hydrogen bonding and electrostatic repulsion, inducing the denaturation and aggregation of surimi proteins to form gels. Second, CO_2_ is a hydrophobic solvent that interacted with the hydrophobic groups in the surimi to induce protein denaturation and aggregation to form gels [32].

**Figure 7:**
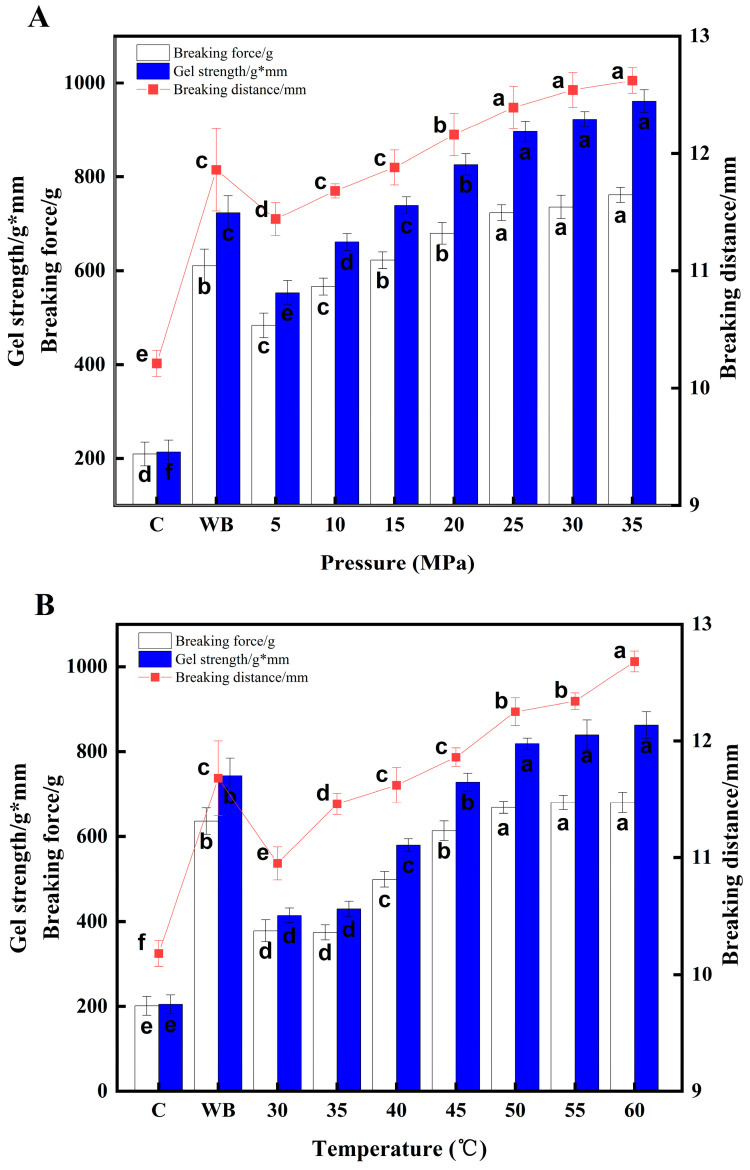

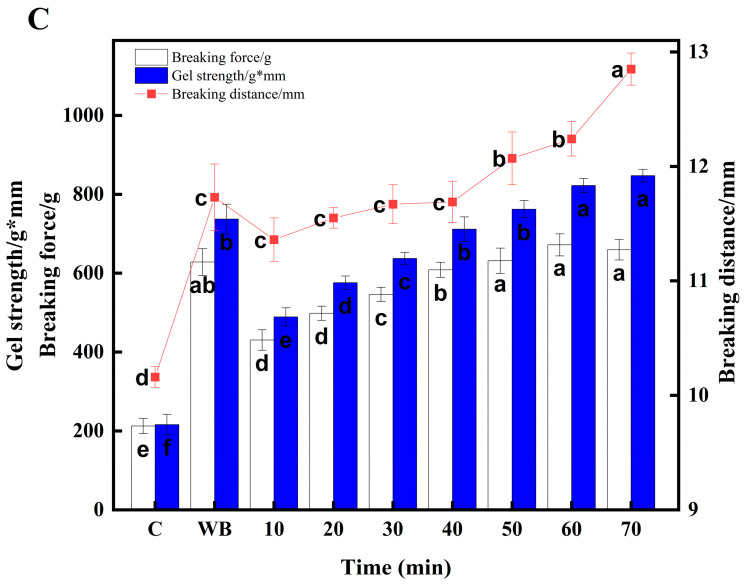
Effect of different DPCD treatments on the surimi gel strength (**A**): Pressure, (**B**): Temperature, (**C**): Time. Different letters indicate statistically significant differences (*p* < 0.05).

The effect of DPCD treatment on the surimi gel strength is shown in Figure 7. Both DPCD treatment and heat treatment significantly increased the surimi gel strength compared to the control group (*p* < 0.05). The gel strength also increased as the intensity of DPCD treatment increased. At DPCD treatment conditions of 50 °C and 60 min (Figure 7A) and treatment pressures in the range of 5–25 MPa, the surimi gel strength increased with increasing pressure (*p* < 0.05). Furthermore, at DPCD treatment pressures of up to 20 MPa, the surimi gel strength was significantly better than that of the samples in the heat treatment group (*p* < 0.05). The increase in pressure increased the CO_2_ density, which in turn increased its permeation. At treatment pressures in the range of 5–15 MPa, protein depolymerisation was low and the internal polar and hydrophobic groups were not fully exposed, preventing the surimi from forming a good gel network structure. However, the surimi gel strength did not change significantly when the treatment pressure reached 25–35 MPa. This showed that when the DPCD treatment conditions were 50 °C, 60 min and 20 MPa, the surimi had formed a good gel structure. Therefore, under these conditions, increasing the treatment pressure had no significant effect on the surimi gel strength.

When the DPCD treatment conditions were 20 MPa and 60 min (Figure 7B) and the treatment temperature was in the range of 30–50 °C, the surimi gel strength increased with increasing temperature *(p* < 0.05). Additionally, the surimi gel strength at DPCD treatment temperatures up to 50 °C was significantly better than that of the samples from the heat treatment group (*p* < 0.05). This was mainly due to the heat-resistant alkaline protease in the surimi, which has an optimum enzymatic activity at 50–70 °C. During heat treatment, proteases degrade myofibrillar proteins, reducing gel elasticity and strength [3]. However, gel degradation was inhibited by DPCD-induced gel formation of the surimi at temperatures below 55 °C and by the passivating effect of DPCD on some heat-tolerant alkaline proteases. However, there was no significant change in the surimi gel strength at DPCD treatment temperatures in the range of 55–60 °C. Furthermore, when the DPCD treatment temperature reached 50 °C, the surimi gel strength was significantly better than that of the heat-treated samples (*p* < 0.05). This showed that a DPCD treatment temperature of 50 °C induced gel network formation at 20 MPa and 60 min; further increasing the treatment temperature did not significantly change the gel strength.

The surimi gel strength increased with time when the DPCD treatment conditions were 20 MPa and 50 °C (Figure 7C) and the treatment times were in the range of 10–60 min. This was due to the cumulative effect of the DPCD treatment on the surimi gel. With increased treatment time, more CO_2_ molecules penetrated the surimi gels and more CO_2_ molecules interacted with the myofibrillar proteins, contributing to the acceleration of the gel network formation. The strength of the surimi gel was significantly better than that of the heat-treated samples (*p* < 0.05) when the DPCD treatment time reached 60 min, indicating that the surimi gel network had formed under these conditions and that there was no significant change in gel strength as the treatment time was further increased.

### 3.5. Correlation between Water Characteristics and Gel Strength during DPCD-Induced Gel Formation in Surimi

To investigate the correlation between the moisture properties and gel strength during DPCD-induced gel formation in golden pompano surimi, a correlation analysis was performed between the above moisture property indicators and gel strength. The correlation heat map was colour-coded with different intensities of blue and red, with blue indicating a negative correlation, red indicating a positive correlation and the intensity of the colour expressed by the Pearson correlation coefficient (P). Depending on the magnitude of P, the correlation was classified as weak (0.0–0.19), medium (0.2–0.6), or strong (0.6–0.1) [11,33].

**Figure 8:**
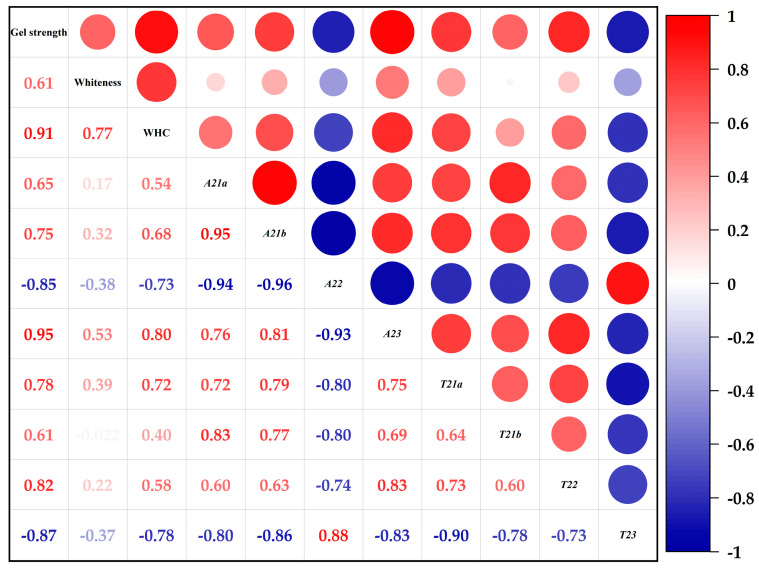
Correlation between water characteristics and gel strength during DPCD-induced gel formation in surimi (WHC: water-holding capacity, *A*_2b1_, *A*_2b2_, *A*_21_, and *A*_22_ represent the contents of strongly bound water, weakly bound water, immobilised water, and free water, respectively, *T*_21*a*_, *T*_21*b*_, *T*_22_ and *T*_23_ represent strongly bound water, weakly bound water, immobilised water, and free water, respectively).

As shown in Figure 8, water-holding capacity, *A*_21a_, *A*_21b_, *A*_23_, *T*_21a_, *T*_21b_ and *T*_22_ were strongly positively correlated with gel strength, while *A*_22_ and *T*_23_ were strongly negatively correlated with gel strength. As the major component of water in surimi, *A*_22_ corresponds to a degree of water mobility that is second only to free water as a layer of immobile water. The decrease in its proportion was to some extent indicative of the overall decrease in water in the surimi gel. Meanwhile, the denaturation of the proteins during gel formation resulted in changes to their hydration, so the increased gel strength inevitably also led to a loss of this water [34,35]. As the component with the longest relaxation time, *T*_23_ corresponds to water molecules with a high degree of freedom. As an increase in gel strength means the formation of a three-dimensional gel network, the gel network has a certain binding effect on this part of the water, decreasing the relaxation time.

## 4. Conclusions

This study showed that DPCD treatment induced gel formation in pompano surimi. Compared to the control group, DPCD treatment had a significant effect on the quality of the surimi gel, yielding a superior product compared to the samples in the conventional heat treatment group. LF-NMR showed that, after DPCD treatment, the relaxation component *T*_22_ of surimi shifted to the right, *T*_23_ shifted to the left, *A*_22_ decreased significantly (*p* < 0.05) and *A*_23_ increased significantly (*p* < 0.05). Correlation analysis showed that the water-holding capacity of surimi was strongly positively correlated with gel strength, while *A*_22_ and *T*_23_ were strongly negatively correlated with gel strength for all DPCD treatment conditions. Therefore, water-holding capacity, *A*_22_ and *T*_23_ can be used as useful indicators to evaluate the changes in gel strength associated with DPCD-induced gel formation in pompano surimi. In future research, a new and rapid gel quality evaluation technique could be developed by modelling based on these moisture characteristics. On this basis, the correlation between gel strength and other microscopic or molecular properties could also be investigated, thereby achieving the rapid and efficient evaluation of surimi gel quality.

## Data Availability

The datasets generated for this study are available upon request to the corresponding author.
